# Is silver diamine fluoride more effective than sodium fluoride varnish at preventing caries in upper anterior primary teeth?

**DOI:** 10.1038/s41432-025-01192-x

**Published:** 2025-11-20

**Authors:** Laura Timms, Zoe Marshman

**Affiliations:** School of Clinical Dentistry, University of Sheffield, Sheffield, UK

**Keywords:** Dentistry, Health care

## Abstract

**A Commentary on:**

**Zheng F M, Yan I G, Duangthip D, Lo E C M, Gao S S, Chu C H**.

Randomized clinical trial on caries prevention of silver diamine fluoride. *J Dent Res* 2025; 10.1177/00220345251363837.

**Design:**

This randomised controlled trial sought to compare the effectiveness of silver diamine fluoride (SDF) to sodium fluoride varnish (NaF) for the prevention of caries in anterior primary teeth for young children. The inclusion criteria were children aged 3–4 years of age. Children were excluded if they had co-operative challenges or were on long-term medications. Children with active caries were included, lesions were considered active if they were soft on probing. The setting was kindergartens in Hong Kong. The children received either 38% SDF or 5% NaF applied to their six upper anterior primary teeth at six-monthly intervals. The primary outcome measure was the mean number of new carious surfaces per child at 30 months follow-up. Intention-to-treat analysis was used with the Mann-Whitney U test applied for the analysis of the primary outcome.

**Results:**

Overall, 730 children were randomised, 621 (85%) were followed up and analysed at the 30 months end point for the presence of new carious surfaces (dmfs). Not all children seen at baseline were analysed, however of those that were their caries experience was similar with a mean dmfs of 0.47 ± 1.35 for the SDF group and 0.48 ± 1.53 for the NaF group. At 30 months, there were less new lesions for those in the SDF group (0.35 ± 1.09), than the NaF group (0.54 ± 1.50) (*p* = 0.048).

**Conclusions:**

The authors concluded that SDF was more effective than NaF in preventing new carious surfaces at 30 months for anterior caries in primary teeth of young children.

## GRADE Rating:



## Commentary

This commentary will focus on the strengths and weaknesses of the trial and the implications for care for young children with caries in their anterior teeth^1^.

While the population in the trial was clearly defined in terms of their age, relevant information not explicit in the inclusion criteria was about caries status, medical history and the children’s level of co-operation. This information would have helped when considering the generalisability of the findings, particularly as children who are unco-operative or on long term medications would benefit from effective prevention.

The sample size calculation was based on reasonable assumptions from previous research on anticipated effect size, using standard values for alpha and beta and two-sided significance. However, the target sample and anticipated loss to follow-up differed from that described in the pre-registered protocol (Clinicaltrials.gov NCT04075474), and this change is not highlighted or explained in the published paper.

The randomisation strategy was appropriate and led to groups that were well balanced at baseline for many important factors including caries status. However, there was some imbalance in the cessation of bottle usage. The authors used a statistical test to compare groups at baseline which is not generally considered appropriate. It would have been useful to know how many children came from each nursery to assess whether this was balanced across the two treatment groups. The research team seemed to consent and carry out baseline measures before revealing which group children had been assigned to, which ensures their allocation was concealed reducing selection bias. Both groups were treated equally throughout the study, other than the preventative treatments that were applied. The application processes of SDF and NaF were clearly described including the specific product, allowing reproducibility of the technique. There was no report of any cross over, and so children were treated in the group they were assigned to, preserving the balance between groups.

While authors report the examiner and families were blinded, this is unlikely to have been feasible in practice. This is owing to the fact SDF when applied to carious lesions changes the lesion colour to black. As children with caries were included, outcome assessors would have been able to tell which group the children had been assigned to, which could introduce performance and detection biases.

There was a clear CONSORT diagram detailing patient flow. The losses to follow-up were identified and reasons provided at each visit. Importantly, children lost to follow-up were not included in the final analysis. Those lost to follow-up had a higher baseline caries experience therefore may have responded differently to the preventative treatments. This could have impacted the findings, although losses were reasonably balanced between the groups. It is interesting to note that the report states all participants co-operated with the interventions however, a small number were excluded owing to lack of co-operation. Clustering of teeth within individuals was accounted for in the analysis.

There was no cost-effectiveness analysis. Given the small increase in benefit for SDF it would be very dependent on the cost of the product within a setting as to whether SDF compared to NaF would likely be cost-effective which would need formal research to assess. This would depend on factors such as the product cost, and the time and resources to apply SDF in the kindergarten setting. This may vary across different settings owing to the products available, as SDF in some countries is much cheaper than others.

While SDF showed benefit over NaF, the absolute difference was small. As well as evidence of effectiveness, there are other important considerations for the implementation of SDF in future as part of an oral health promotion programme in nurseries and schools. Firstly, SDF is not licensed for caries prevention in many countries which would limit its use if a licenced product such as NaF is available. Secondly, in this trial there was not a formal assessment of harms. SDF has known side effects which include discolouration of carious lesions, potential for soft tissue staining and soft tissue burn with some products where there is prolonged contact with the soft tissues^2^. These features of the use of SDF in an education setting without the availability of a dental unit are significant. In terms of SDF’s discolouration of carious lesions, there is less parental acceptance of SDF on anterior teeth than posterior teeth^3^ so it would be anticipated that providing information to parents and gaining their consent could be difficult. Finally, when administering a topical preventative fluoride preparation, either in a clinical or education setting, applying it to the whole dentition in one visit is beneficial. For SDF, based on the manufacturers advice on the quantities of SDF to be used at one visit there would be a need to apply the solution over multiple time points, again with implications for the practicability and cost-effectiveness.

Overall, while this trial was generally well conducted and reported, there are wider considerations for the implementation of an intervention such as SDF than effectiveness.

Practice Point

- While SDF showed benefit over NaF, the absolute difference was small. As well as evidence of effectiveness, there are other important considerations for the implementation of SDF in future as part of an oral health promotion programme in nurseries and schools.
